# *TP53* and *KRAS* co-mutations are associated with worse outcomes in mucinous ovarian carcinomas

**DOI:** 10.3389/fonc.2025.1573801

**Published:** 2025-08-14

**Authors:** Yingao Zhang, Savannah Barkdull, Panayiotis D. Kontoyiannis, Alejandra Flores Legarreta, David M. Gershenson, Preetha Ramalingam, Michael M. Frumovitz, Anil K. Sood

**Affiliations:** ^1^ Department of Obstetrics & Gynecology, Baylor College of Medicine, Houston, TX, United States; ^2^ McGovern Medical School, University of Texas Health Science Center, Houston, TX, United States; ^3^ Department of Gynecologic Oncology and Reproductive Medicine, University of Texas Medical Doctor (MD) Anderson Cancer Center, Houston, TX, United States; ^4^ Department of Anatomical Pathology, University of Texas Medical Doctor (MD) Anderson Cancer Center, Houston, TX, United States

**Keywords:** mucinous adenocarcinoma, ovarian cancer, *TP53*, molecular sequencing, NGS, genomic sequencing, tumor mutation

## Abstract

**Objective:**

Mucinous ovarian carcinomas (mOC) often harbor unique molecular alterations differentiating them from other epithelial ovarian carcinoma subtypes. We sought to characterize the somatic genomic mutation patterns in mOC and elucidate their associations with oncologic outcomes.

**Methods:**

All patients with mOC treated at a single institution between 2005–2023 were identified, and those with validated tumor molecular profiling (TMP) using next-generation sequencing of somatic variants were included. Progression-free survival (PFS) and overall survival (OS) were calculated on a Kaplan-Meier estimator. Multivariable analysis was performed using Cox regression models.

**Results:**

Forty patients were included in this retrospective cohort; 34 (85%) had at least 1 genomic alteration on TMP, with a median of 3 mutations (range 0-30). *TP53* (68%) and *KRAS* (63%) were most frequently altered, and 21 patients (53%) had tumors with *TP53*/*KRAS* co-mutations. Patients with *TP53*/*KRAS* co-mutations were younger (median 27.9 vs 54.1 y, p=0.01) and were more likely to have early-stage disease (86% vs 47%, p=0.02) than patients without these co-mutations. On multivariable analysis, *TP53*/*KRAS* co-mutations were associated with decreased PFS (adjusted hazard ratio [aHR] 4.02, 95% confidence interval [CI] 1.46-12.5, p=0.01) and OS (aHR 21.4, 95% CI 4.28-156, p<0.001). On subgroup analysis of stage I tumors (N=27), the presence of *TP53*/*KRAS* co-mutations remained independently associated with worse OS (aHR 8.66, 95% CI 1.50-93.8, p=0.03).

**Conclusion:**

A substantial proportion of mOCs have concurrent *TP53* and *KRAS* alterations on TMP, and this may portend worse survival, even for patients with early-stage disease. TMP could be a useful tool for prognostication and can be considered for patients with mOC at the time of diagnosis.

## Introduction

Ovarian cancer is a leading cause of mortality in patients with gynecologic malignancies and the second most common type of gynecologic malignancy in the United States (1). Mucinous ovarian carcinoma (mOC) is a rare subtype of epithelial ovarian carcinoma that accounts for less than 5% of all ovarian cancer cases (2, 3). Traditionally, the mainstay of treatment is surgery followed by chemotherapy for patients with advanced-stage or higher-risk disease. The 5-year overall survival of patients who present with early-stage disease usually exceeds 80% but drastically decreases for those with advanced-stage disease (3). When compared to high-grade serous ovarian carcinomas, mOC is less responsive to traditional cytotoxic chemotherapy; additionally, there is a lack of consensus on the preferred treatment regimen (4).

Due to these challenges and the unique behavior of mOC, researchers and clinicians have sought to characterize its distinct molecular profile in the hopes of better understanding its pathogenesis and identifying possible therapeutic targets. *KRAS* and *TP53* gene mutations are commonly implicated in many types of cancer, including epithelial ovarian cancer (5). *KRAS* is a proto-oncogene that functions as a key mediator of the RAS signaling pathway, which drives cell growth and proliferation; specifically, codon 12 is recognized as a mutational “hotspot” across many cancer types. *TP53* is a tumor suppressor gene commonly mutated in a variety of cancers; its mutation leads to a variety of genetic aberrations that can lead to cancer formation and progression. The most common alterations in *TP53* include missense and nonsense mutations, many of which impair its DNA binding and transactivation functions (6, 7). In this study, we sought to characterize the mutational landscape of mOCs from patients treated at our institution and to examine potential associations of *TP53* and *KRAS* mutations with patient outcomes.

## Methods

Following IRB approval, a retrospective cohort study of all patients diagnosed with mOC between 2005–2023 at a single comprehensive cancer center was performed. Study subjects were identified as part of an institutional internal rare cancers’ registry, and histology was confirmed by central pathology review. Patients were included if they had tumor molecular profiling (TMP) via next-generation sequencing (NGS). Patients were excluded if surgery was not performed at some point during their treatment, if they had mixed histology or mOC from a separate site, a synchronous malignancy, or an incomplete medical record available for review (**Supplementary Figure 1.1**).

Basic demographic information was extracted from the electronic medical record. Cancer-specific data were also abstracted, including stage, grade, surgical records, all TMP and genomic data, as well as dates of diagnosis, treatment, recurrence, and death. TMP and genomic data were obtained from either internal or external sequencing panels, which were ordered either at initial diagnosis or at the time of recurrence, based on the discretion of the primary treating oncologist. All pathogenic variants identified through these panels were recorded (**Supplementary Table 1.2**). Individual mutations in *TP53* and *KRAS* were then independently cross-referenced with ClinVar and the NCI’s *TP53* Database to assess and validate their functional significance. Study data were collected and managed using REDCap (Research Electronic Data Capture) hosted at our institution. Staging was based on FIGO 2014 criteria, and all cases diagnosed prior to 2014 were “re-staged” for the purposes of this study. Primary outcomes included progression-free survival (PFS) and overall survival (OS). PFS was defined as the length of time from the date of diagnosis to the date of disease recurrence, and OS was defined as the length of time from the date of diagnosis to the date of death. Patients were censored if they did not experience disease recurrence or death by the end of data collection (December 31, 2023).

Demographic and clinical characteristics were analyzed and compared using t-tests and chi-square/Fisher exact tests. Survival indices were calculated on a Kaplan-Meier estimator using the log-rank method. Multivariable analyses were performed using Cox regression models. All statistical analyses were performed using GraphPad Prism 10.2.1 (Dotmatics; Boston, MA, USA). Statistical significance was set at p ≤ 0.05.

## Results

**Figure 1:**
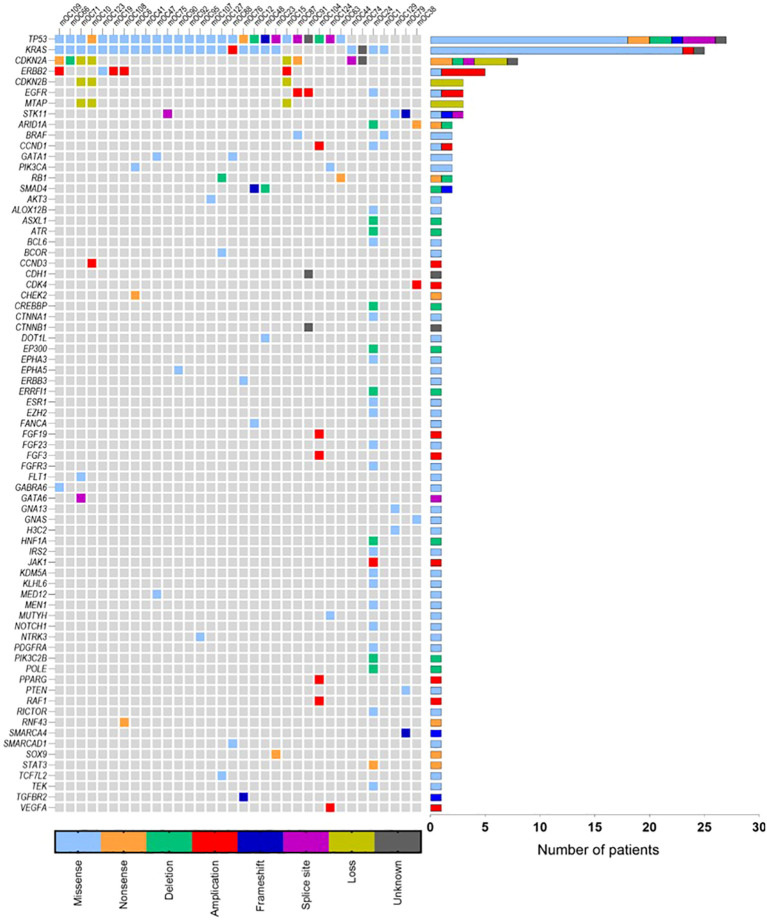
Oncoplot of mOC patients with alterations (N=34) identified on NGS. Six patients without any identifiable mutations are not included. *TP53* (N=27) and *KRAS* (N=25) were the most frequently identified genes with alterations. Missense mutations were the most common detected type (56.4%), followed by amplifications (10.7%) and deletions (9.4%).

A total of 40 patients were included in the final cohort (**Supplementary Table 1.1**). All patients underwent primary surgery, and 25 (63%) received adjuvant systemic chemotherapy. At least 1 genomic alteration was identified on TMP in 34 patients (85%), with a median of 3 mutations (range 0-30). In total, 148 alterations in 73 different genes were found, with missense mutations being the most common (55.9%), followed by amplification (10.5%) and deletion (8.6%) (**Figure 1**). *TP53* (67.5%) and *KRAS* (62.5%) were the most frequently altered genes; 21 patients (53%) had tumors with *TP53*/*KRAS* co-mutations. Most KRAS mutations occurred at codon 12, with G12D (44%) and G12V (32%) being the most prevalent. TP53 mutations were more diverse, with 23 distinct variants identified (**Supplementary Table 1.3**).

**Figure 2:**
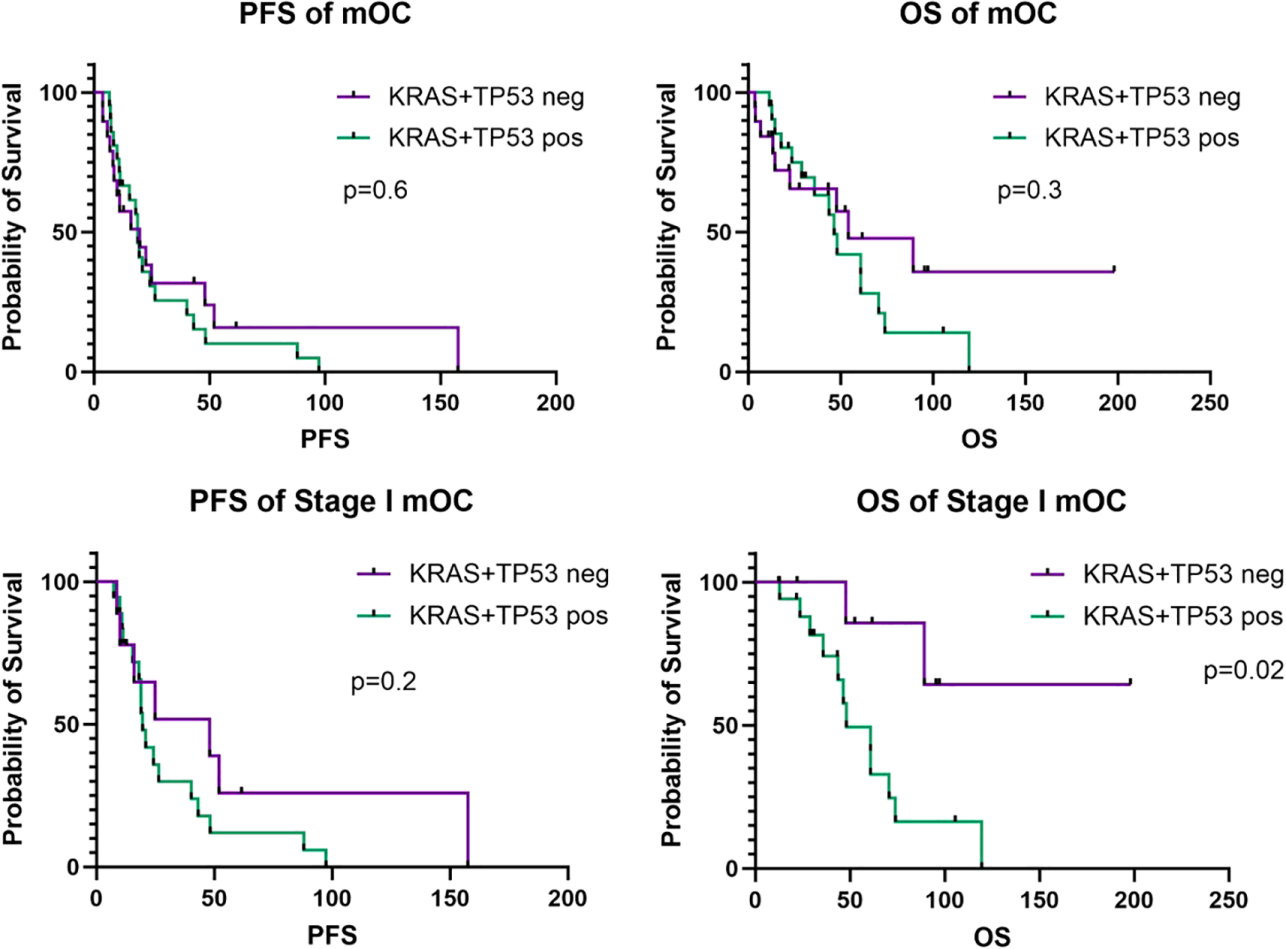
Progression-free survival (PFS) and overall survival (OS) curves for the entire study cohort (N=40) and the stage I subgroup (N=27). Survival analyses were calculated on a Kaplan-Meier estimator using the log-rank method. Y-axis (probability of survival) is quantified by percentages. X-axis (PFS or OS) is quantified by number in months. mOC, mucinous ovarian cancer.

Patients with *TP53*/*KRAS* co-mutations were younger (median 27.9 vs 54.1 y, p=0.01) and were more likely to have stage I disease (86% vs 47%, p=0.02) than patients without *TP53*/*KRAS* co-mutations (**Table 1A**). There were no differences in body mass index (BMI), tumor grade, or chemotherapy administration between the 2 groups. Samples collected from recurrent tumors demonstrated more *TP53*/*KRAS* co-mutations than did primary tumor samples (62% vs 38%), but this difference was not significant (p=0.07). On subgroup analysis of patients with stage I disease, there were no differences in age at diagnosis, BMI, tumor grade, chemotherapy administration, or TMP timing (**Table 1B**). There were also no differences in the proportion of patients with stage IA or IC disease at diagnosis (p>0.9). In this analysis, stage IC included IC1 (N=8), IC2 (N=1), and IC3 (N=7); there were no patients with stage IB disease at diagnosis. In the overall cohort, patients with *TP53*/*KRAS* co-mutations and those without did not have significantly different median PFS (18.9 vs 19.8 months, p=0.6) or OS (46.5 vs 54.0 months, p=0.3); however, among patients with stage I disease, those with *TP53*/*KRAS* co-mutated tumors had significantly shorter median OS compared to those without the co-mutation (47.9 months vs median not met, p=0.02) (**Figure 2**). No significant differences in survival curves were observed when stratifying by individual mutations in either *KRAS* or *TP53* alone (**Supplementary Figures 1.2 & 1.3**).

**Table 1A:** Comparison of demographic and clinical characteristics between patients with and without KRAS/TP53 co-mutations.

		*TP53*_wt_ or *KRAS*_wt_ (n=19)	*KRAS*- & *TP53*-altered (n=21)	p-value
**Age, median, y**		**54.1**	**27.9**	**0.01**
**BMI, median, kg/m^2^**		27.0	25.4	0.3
**Stage**				**0.02**
	I	**9 (47)**	**18 (86)**	
	II/III/IV	**10 (53)**	**3 (14)**	
**Grade**				0.5
	1	7 (37)	5 (24)	
	2	8 (42)	13 (62)	
	3	4 (21)	3 (14)	
**Timing of TMP**				0.07
	Primary	13 (68)	8 (38)	
	Recurrence	6 (32)	13 (62)	
**Adjuvant chemotherapy**		14 (74)	11 (52)	0.2
**PFS, median, months**		19.8	18.9	0.6
**OS, median, months**		54.0	46.5	0.3

**Table 1B:** Stage I cohort.

		*TP53*_wt_ or *KRAS*_wt_ (n=9)	*KRAS*- & *TP53*-altered (n=18)	p-value
**Age, median, y**		38.2	36.4	0.4
**BMI, median, kg/m^2^**		26.4	25.4	0.2
**Stage**				>0.9
	IA	4 (44)	7 (39)	
	IC	5 (56)	11 (61)	
**Grade**				0.1
	1	5 (56)	3 (17)	
	2	3 (33)	12 (67)	
	3	1 (11)	3 (17)	
**Timing of TMP**				0.7
	Primary	5 (56)	8 (44)	
	Recurrence	4 (44)	10 (56)	
**Adjuvant chemotherapy**		4 (44)	8 (44)	>0.9
**PFS, median, months**		48.0	19.6	0.2
**OS, median, months**		**Not met**	**47.9**	**0.002**

Data are shown as no. of patients (%) unless otherwise indicated. Statistical significance was determined using Student t-tests and Fisher exact tests. Statistically significant values are bolded. BMI = body mass index; mOC = mucinous ovarian cancer; PFS = progression free survival; OS = overall survival; TMP = tumor molecular profiling.

Statistically significant values are bolded.

Clinically relevant variables were selected as potential confounders. After adjusting for stage and age, as well as grade, chemotherapy, and timing of TMP collection, multivariable analyses demonstrated that the presence of *TP53*/*KRAS* co-mutations was associated with both lower PFS (aHR 4.02, 95% CI 1.46-12.5, p=0.01) and OS (aHR 21.4, 95% CI 4.28-156, p<0.001) (**Table 2A**). Other independent associations found were tumor grade with worse OS (adjusted hazard ratio [aHR] 2.19, 95%CI 1.10-4.29, p=0.02), and stage with both worse PFS (aHR 7.42, 95% CI 2.55-23.1, p<0.001) and OS (aHR 11.6, 95% CI 3.12-50.0, p<0.001). On subgroup analysis of stage I tumors (N=27), the presence of *TP53*/*KRAS* co-mutations remained independently associated with worse OS (aHR 8.66, 95% CI 1.50-93.8, p=0.03) (**Table 2B**). High-grade disease was also independently associated with worse OS (aHR 2.53, 95% CI 1.02-6.74, p=0.05). Substage (stage IA vs IC) was not associated with changes in either PFS or OS.

**Table 2:** Multivariable analyses of independent associations of clinical factors and KRAS/TP53 mutation status with survival. Full study cohort (N=40).

		PFS			OS		
		aHR	95% CI	p-value	aHR	95% CI	p-value
Age at diagnosis		1.02	0.99-1.04	0.1	1.02	0.99-1.06	0.2
Stage		**7.42**	**2.55-23.1**	**<0.001**	**11.6**	**3.12-50.0**	**<0.001**
Grade		1.58	0.90-2.71	0.1	**2.19**	**1.10-4.29**	**0.02**
Chemotherapy		0.40	0.14-1.06	0.07	0.63	0.18-2.12	0.5
TMP Timing		1.18	0.55-2.53	0.7	1.05	0.39-2.85	0.9
Mutations							
	# Detected	0.95	0.87-1.01	0.1	0.94	0.80-1.03	0.3
	KRAS	1.37	0.62-3.29	0.5	2.29	0.82-7.68	0.8
	TP53	**3.66**	**1.33-11.2**	**0.02**	**7.95**	**2.06-37.9**	**0.005**
	KRAS & TP53	**4.02**	**1.46-12.5**	**0.01**	**21.4**	**4.28-156**	**<0.001**

**Table 2B:** Stage I cohort (n=27).

		PFS			OS		
		aHR	95% CI	p-value	aHR	95% CI	p-value
Age at diagnosis		1.03	0.99-1.06	0.1	1.02	0.97-1.06	0.5
Substage		1.21	0.49-3.05	0.7	2.24	0.67-8.52	0.2
Grade		1.48	0.71-3.13	0.3	**2.53**	**1.02-6.74**	**0.05**
Chemotherapy		0.74	0.25-2.11	0.6	0.48	0.10-2.21	0.3
TMP Timing		1.26	0.46-3.51	0.7	1.19	0.29-5.50	0.8
Mutations							
	# Detected	1.41	0.93-2.21	0.1	1.45	0.90-2.56	0.2
	KRAS	0.77	0.26-2.48	0.6	1.69	0.36-12.8	0.5
	TP53	1.21	0.34-5.06	0.8	**13.0**	**1.45-334**	**0.05**
	KRAS & TP53	1.06	0.34-3.61	0.9	**8.66**	**1.50-93.8**	**0.03**

Cox regression analyses were used to control for age at diagnosis, stage, grade, receipt of chemotherapy, and timing of TMP, and the Bonferroni adjustment was used to correct for multiple comparisons. Statistically significant values are bolded. aHR = adjusted hazard ratio; CI = confidence interval; PFS = progression free survival; OS = overall survival; TMP = tumor molecular profiling.

Statistically significant values are bolded.

## Discussion

For early-stage mOC, comprehensive surgery can be curative without the need for adjuvant treatment, with reported 5-year survival rates of 86-90% (8, 9). For patients with advanced-stage or recurrent disease, however, there is a paucity of evidence to guide treatment owing to the rarity of this tumor. There is also no clear consensus on how to appropriately risk-stratify patients with early stage mOC for the need for adjuvant chemotherapy. Current guidelines are extrapolated from historical trials dominated by other histological subtypes of high-grade epithelial carcinomas (10). There is growing evidence that, much like low-grade serous ovarian cancer, mucinous tumors represent a separate and distinct entity from other epithelial ovarian malignancies with respect to their pattern of invasion, recurrence, and response to therapy (11, 12).

At present, the extent of disease warranting adjuvant treatment for stage I mOC is up for debate, with some groups advocating more aggressive treatment starting with stage IB rather than stage IC disease (13, 14). There is less dispute about the management of stage IA disease with surgery alone, though some recent evidence suggests that consideration of adjuvant treatment for patients with infiltrative subtypes may be reasonable given the potentially higher risk of recurrence (15–17). Retrospective cohorts have shown that patients with infiltrative stage I disease demonstrate increased rates of recurrence when compared to those with expansile histology, especially after fertility-sparing surgery, and that survival outcomes were comparable to patients with stage I high grade serous carcinomas (18, 19).

Furthermore, the choice of specific adjuvant therapies remains unsettled. Following the early closure of GOG241 due to poor accrual, some retrospective data have suggested the use of a gastrointestinal-based regimen of oxaliplatin, capecitabine, and 5-fluorouracil over the traditional ovarian cancer-based carboplatin and paclitaxel regimen, but clear survival benefit has not been consistently demonstrated (20–22).

Our data suggest that tumor mutational status could be an important step in risk stratification, as patients with *TP53*/*KRAS* co-mutations had significantly shorter OS than did patients without co-mutations, particularly among those with stage I disease at diagnosis. Given that both stage I subgroups had otherwise similar clinical and demographic features, it is possible that the distinct genetic profiles could be driving their disparate outcomes. These results may warrant consideration of collecting TMP for patients with mOC.

In addition to determining who needs adjuvant treatment, there is also a need for targeted treatment options more suited to the distinct molecular profile of mOC. As TMP becomes more widely implemented in oncology, specific actionable genetic targets have been identified. In *KRAS*-altered carcinomas and adenocarcinomas, codon 12 is the most frequently mutated, and the G12D mutation is generally the most prevalent (23). Its favorable structural conformation and downstream targeting has been shown to create protein expression profiles distinct from other *KRAS* mutations, contributing to its high oncogenic potential as well as unique clinical outcomes (24). In our cohort, the G12D mutation was present in most of the *KRAS*-altered tumors, consistent with its reported prevalence in other *KRAS*-altered cancer types. In contrast, *TP53* mutations exhibit a much wider heterogeneity in mOC, making therapeutic targeting more challenging in this population (**Supplementary Table 1.3**).

Recent breakthroughs in targeting the *RAS* oncogene began with KRAS-G12C inhibition, which has shown some promise in phase I/II clinical trials for metastatic lung, pancreatic, and colorectal carcinomas (25–28). Small molecules with anti-KRAS-G12D activity have shown remarkable response in preclinical models, and several first-in-human trials are currently in recruitment, with clinical data anticipated as early as this year (NCT05533463, NCT05737706, NCT06040541, NCT05382559) (29–34). It is important to note that though these trials are open to patients with all solid tumors with the KRAS-G12D mutation, their supporting preclinical data rarely include gynecologic cancer models. As underscored by our cohort, a substantial proportion of mOCs harbor *KRAS* mutations. The negative associations of *KRAS* mutations with recurrence and survival are not limited to mOC and are evident in other subtypes of epithelial ovarian cancers as well (35, 36). Additionally, somatic mutational analysis comparing mOC with mucinous carcinomas of other primary origins showed significant parallels between mOC and pancreatic carcinomas, especially with respect to specific *KRAS* and *TP53* mutations (37). This may represent a potential alternative or subsequent therapy to traditional chemotherapy for patients with mOC, especially in the contemporary era of increasing approvals for mutation-driven, tumor-agnostic treatment options (38–40).

This study is limited by its retrospective and single-institutional nature. Though this work represents one of the largest single institutional retrospective cohorts reported for mOC, statistical associations should be carefully interpreted in the context of the relatively small sample size and limited power, especially evident in some of the wide confidence intervals in this analysis. Furthermore, the 18-year duration of the study coincided with significant advances in sequencing technology, including improvements in accessibility, sensitivity, and throughput, both within our institution and across commercial platforms. However, specific details regarding the evolution of “generations” of testing used by external companies are not publicly available (**Supplementary Table 1.2**). Although the availability of molecular profiling was clearly beneficial for patient care, the use of heterogeneous sequencing platforms introduces the potential for unrecognized confounding variables, particularly in a retrospective study where testing methodologies varied over time.

In this cohort, the histologic distinction between infiltrative and expansile patterns was only available for a small portion of our patients, and thus not included in the analysis; recent retrospective survival data suggest that this classification may be a major confounding variable and should remain an active area of focus in future studies.

While our findings demonstrated a survival difference, the current data are not of sufficient quality to support changes in the treatment paradigm for patients with early-stage mOC. We hope that our genomic data contribute meaningfully to the existing literature and may assist clinicians in risk-stratification when counseling patients who have undergone tumor molecular profiling. However, we emphasize that the evidence remains insufficient to justify treatment decisions based solely on mutational status. Larger-scale prospective studies are needed to validate these associations, though their feasibility is limited by the rarity of this disease. Ongoing and future tumor-agnostic biomarker-driven trials offer more immediate clinical relevance for the mOC treatment landscape.

The broader implementation of TMP for patients with mOC is not without significant challenges. Despite substantial advances in sequencing technology and gradual improvements in accessibility and insurance coverage over the past two decades, molecular profiling remains underutilized, especially outside of major academic centers. In the United States, many initial surgeries are performed by general gynecologists for presumed benign masses, and patients are referred to oncologists only after cancer is found on final pathology. For patients whose original tissue blocks are not available for testing, circulating tumor DNA (ctDNA) could serve as a proxy for any minimal residual disease left after primary surgery. CtDNA-based genomic sequencing has shown potential for predicting recurrence and guiding adjuvant therapy decisions in several solid tumors, including breast, colorectal, and lung cancers (41). Moreover, multiple studies have demonstrated that ctDNA is a viable marker for identifying specific mutations, estimating disease burden at diagnosis, and monitoring treatment response in epithelial ovarian cancer (42–44). Although further validation is needed, ctDNA remains a promising adjunct—and in some cases, a potential alternative—to traditional TMP in mOC and other gynecologic malignancies.

Our study underscores the importance of molecular profiling in mOCs, especially in patients with early-stage disease. We propose that universal TMP should be considered for all cases of mOC—when available and financially accessible—given its potential prognostic value and possibility of informing future shifts in treatment strategies. While large-scale, prospective trials in mOC will continue to pose logistical challenges due to the rarity of the disease, they may become feasible through coordinated multi-institutional collaboration.

## Author’s note

This abstract was presented at the Society for Gynecologic Oncology Annual Meeting for Women’s Cancer: March 16-18, 2024, San Diego, California, USA.

## Data Availability

The raw data supporting the conclusions of this article will be made available by the authors, without undue reservation.
