# Supplementary material for: TP53 and KRAS co-mutations are associated with worse outcomes in mucinous ovarian carcinomas
Source: Front Oncol. 2025 Aug 14;15:1573801. doi: 10.3389/fonc.2025.1573801 (PMC12390820; doi:10.3389/fonc.2025.1573801)
Supplement: Supplementary file 1 [file DataSheet1.docx]

Supplementary Figure 1.1 – Patient cohort inclusion and exclusion flowchart. LMP, low malignant potential; TMP, tumor molecular profiling


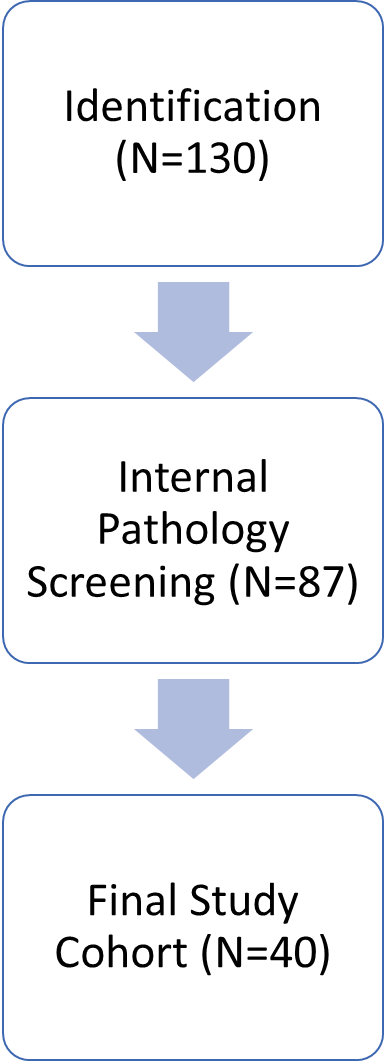


Exclusions:

- Other concurrent malignancies with overlapping treatments (N=6)

- No TMP completed (N=41)

Exclusions:

- LMP/borderline histology (N=14)

- Other (i.e. high grade serous) or mixed ovarian histology (N=11)

- Mucinous malignancy of non-ovarian primary (N=18)

Supplementary Table 1.1 – Patient demographic and basic clinical data. Total patient cohort, N=40.

|  | | **Number of patients (%)**  **(N=40)** |
| --- | --- | --- |
| Age at diagnosis, y | |  |
|  | Median | 41.9 |
|  | IQR | 27.5-57.2 |
| Race | |  |
|  | White | 30 (75) |
|  | African American | 2 (5) |
|  | Asian | 6 (15) |
|  | Other/unspecified | 2 (5) |
| Ethnicity | |  |
|  | Hispanic | 3 (8) |
|  | Non-Hispanic | 37 (93) |
| BMI (median, kg/m^2^) | | 25.5 |
| CA-125 at diagnosis, U/mL | |  |
|  | Median | 89.5 |
|  | IQR | 27.0-241.7 |
| CEA at diagnosis, ng/mL | |  |
|  | Median | 2.8 |
|  | IQR | 1.3-4.5 |
| Surgery | |  |
|  | Laparotomy | 28 (65) |
|  | Laparoscopy | 6 (15) |
|  | Robotic-assisted | 6 (15) |
|  | Conversion to laparotomy | 2 (5) |
| Stage | |  |
|  | IA | 11 (28) |
|  | IC1 | 8 (20) |
|  | IC2 | 1 (3) |
|  | IC3 | 7 (18) |
|  | IIB | 1 (3) |
|  | IIIA1 | 2 (5) |
|  | IIIB | 1 (3) |
|  | IIIC | 7 (18) |
|  | IVA | 2 (5) |
| Grade | |  |
|  | 1 | 12 (30) |
|  | 2 | 21 (53) |
|  | 3 | 7 (18) |
| Tumor size, largest dimension, median, cm | | 20.3 |
| Adjuvant treatment | |  |
|  | Systemic chemotherapy | 25 (63) |
|  | No chemotherapy | 15 (38) |
| TMP obtained | |  |
|  | At primary diagnosis | 21 (53) |
|  | At recurrence | 19 (48) |
| Mutation detected | | 34 (85) |
|  | *TP53* | 27 (68) |
|  | *KRAS* | 25 (63) |
|  | *TP53* & *KRAS* | 21 (53) |

Supplementary Table 1.2 – Tumor molecular profiling details

|  | | **Number of patients (N=40)** | **Number of genes tested** |
| --- | --- | --- | --- |
| Sequencing panel | |  |  |
|  | MDA MAPP^§^ | 21 | 610/146 |
|  | Foundation One | 8 | 324 |
|  | Caris | 5 | 592 |
|  | Tempus | 3 | 648 |
|  | Strata | 2 | 429 |
|  | Myriad | 1 | 523 |

^§^MDA MAPP (MD Anderson Mutation Analysis Precision Panel) is an institutional custom high-throughput chemiluminescent immunoassay. In this cohort, the first-generation panel, which included 146 genes, was used for patients who underwent testing before 2021 (N=9). Subsequent patients received testing with the updated panel (N=12), which included 610 genes. *KRAS* and *TP53* were included on all panels listed above.

Supplementary Table 1.3 – Breakdown of specific *KRAS* and *TP53* mutations

|  | | **Number of patients (N=40)** |
| --- | --- | --- |
| *KRAS* (N=25) | |  |
|  | G12D | 11 |
|  | G12V | 8 |
|  | G12A | 1 |
|  | G12R | 1 |
|  | G12 CNV | 1 |
|  | Q61H | 2 |
|  | Unknown / unspecified | 1 |
| *TP53* (N=27) | |  |
|  | C176Y | 2 |
|  | R175H | 2 |
|  | R282W | 2 |
|  | 376-2A | 1 |
|  | 559-1G | 1 |
|  | 673-2A | 1 |
|  | A276G | 1 |
|  | C242Y | 1 |
|  | F109S | 1 |
|  | H179R | 1 |
|  | I255* | 1 |
|  | L130V | 1 |
|  | N200fs | 1 |
|  | Q167P | 1 |
|  | R196* | 1 |
|  | R213* | 1 |
|  | R248Q | 1 |
|  | R273C | 1 |
|  | R273H | 1 |
|  | R306* | 1 |
|  | R337C | 1 |
|  | S215N | 1 |
|  | Y220C | 1 |
|  | Unknown / unspecified | 1 |

Supplementary Figure 1.2 – Survival curves and indices for single KRAS and TP53 mutations. Y-axis (probability of survival) is quantified by percentages. X-axis (PFS or OS) is quantified by number in months.


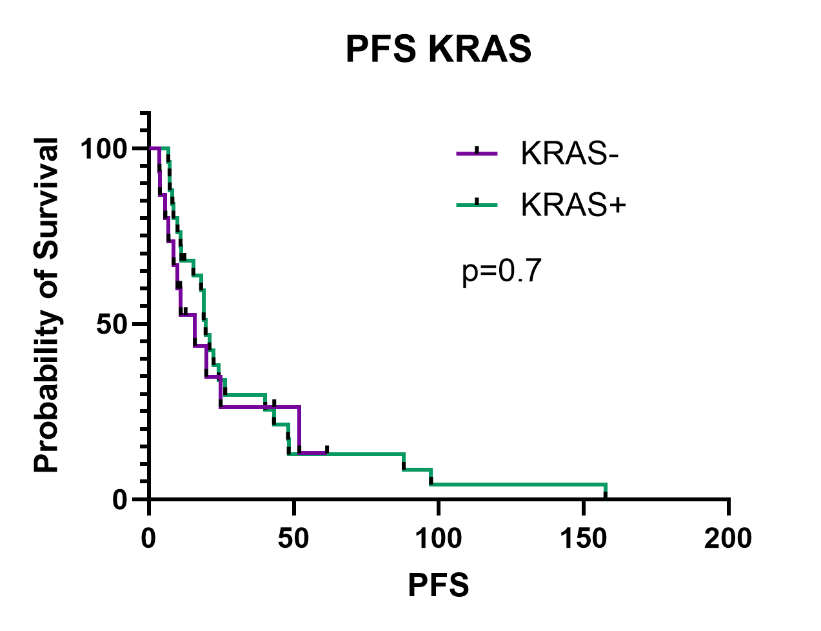

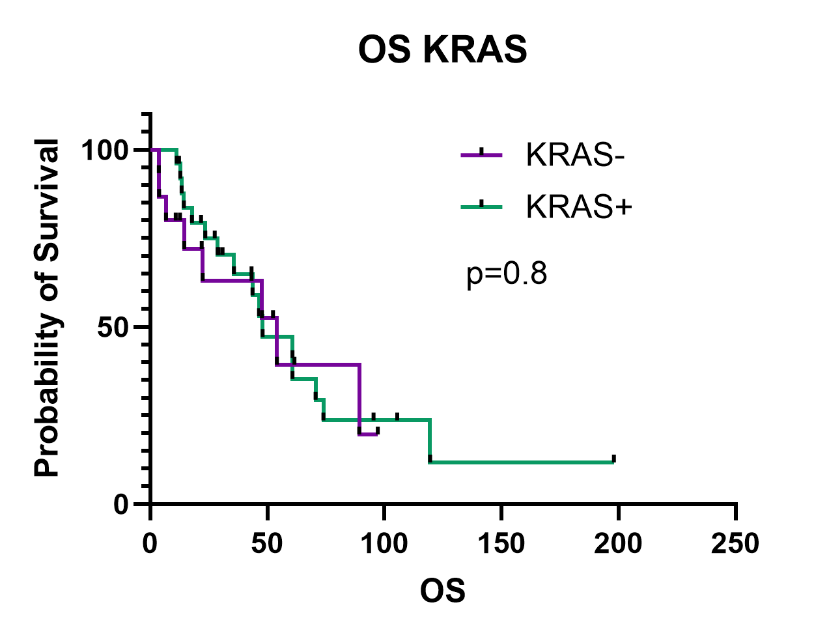


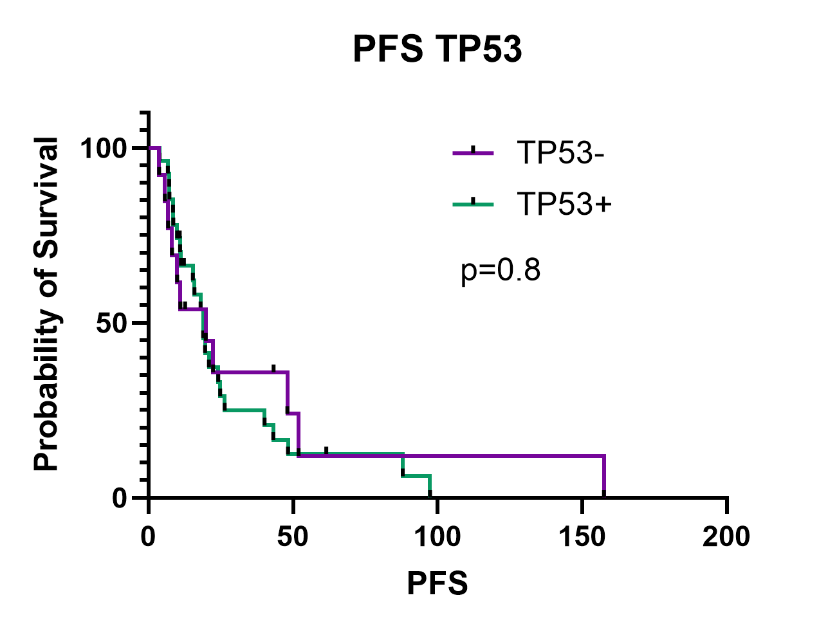

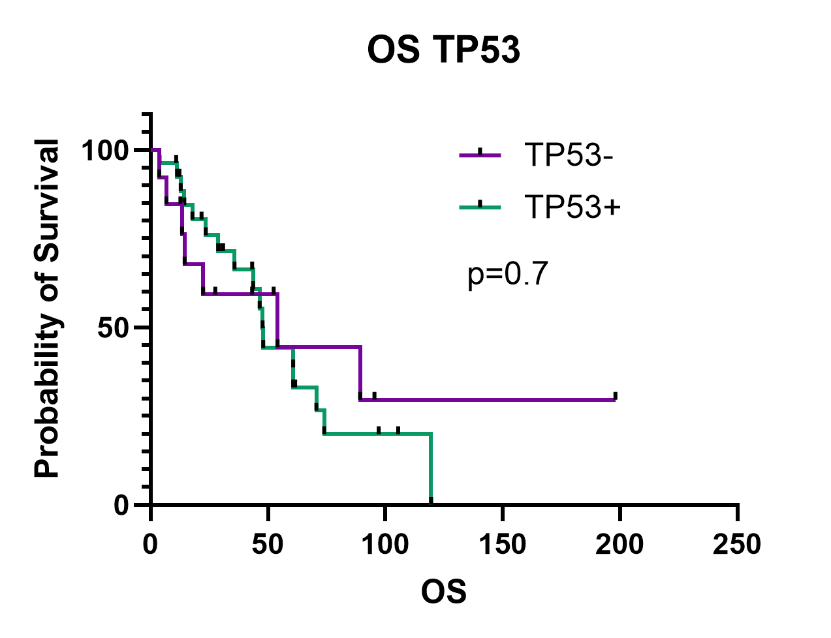


|  | **KRAS+** | **KRAS-** | **TP53+** | **TP53-** |
| --- | --- | --- | --- | --- |
| PFS (median, months) | 19.6 | 15.9 | 18.9 | 19.8 |
| OS (median, months) | 47.9 | 54.0 | 47.7 | 54.0 |

Supplementary Figure 1.3 – Survival curves and indices for single KRAS and TP53 mutations in stage I patients


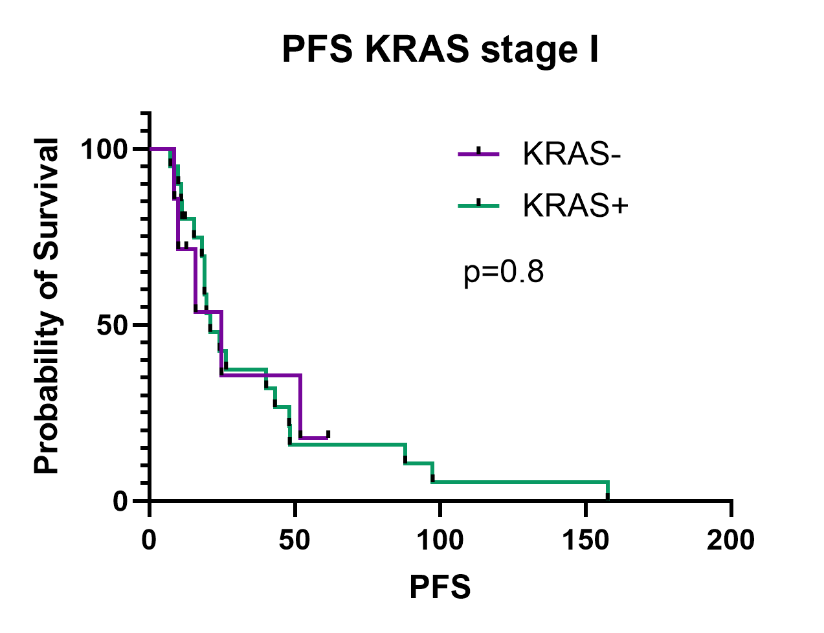

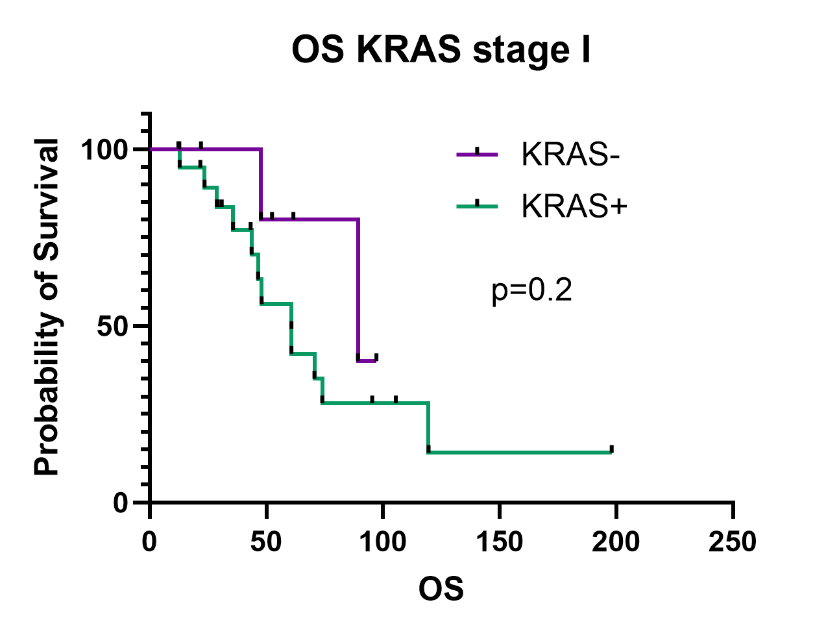


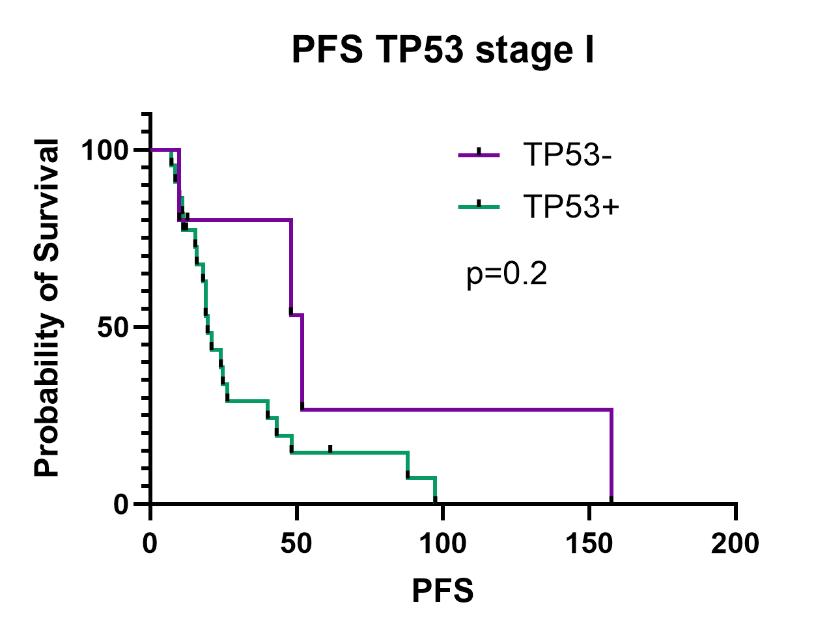

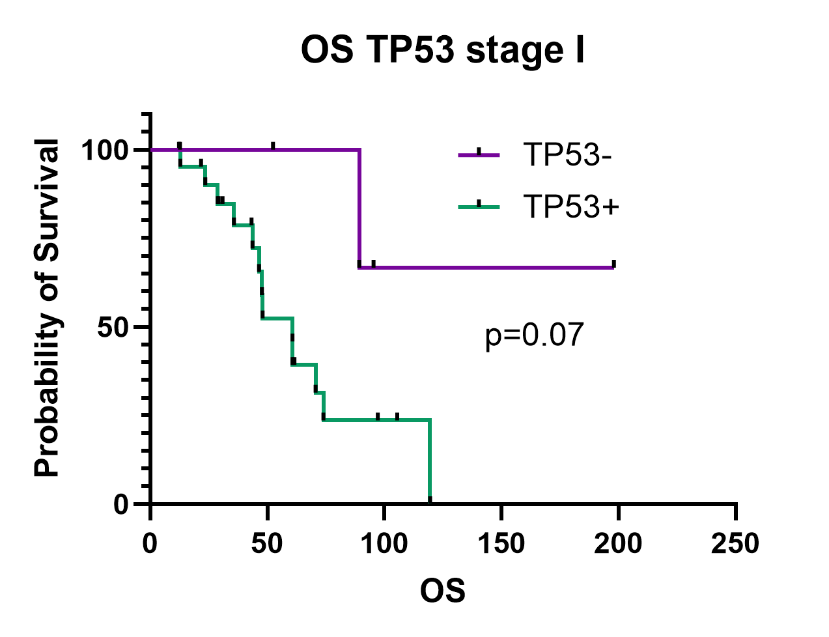


|  | **KRAS+** | **KRAS-** | **TP53+** | **TP53-** |
| --- | --- | --- | --- | --- |
| PFS (median, months) | 20.9 | 24.8 | 19.6 | 51.9 |
| OS (median, months) | 60.8 | 89.3 | 60.8 | not met |
